# Clinical variables associated with late-onset thrombotic and cardiovascular events, after SARS-CoV-2 infection, in a cohort of patients from the first epidemic wave: an 18-month analysis on the “Surviving-COVID” cohort from Bergamo, Italy

**DOI:** 10.3389/fcvm.2023.1280584

**Published:** 2023-11-30

**Authors:** S. V. Benatti, S. Venturelli, G. Crotti, A. Ghirardi, F. Binda, M. Savardi, G. Previtali, M. Seghezzi, R. Marozzi, A. Corsi, P. A. Bonaffini, M. Gori, A. Falanga, A. Signoroni, M. G. Alessio, A. Zucchi, T. Barbui, M. Rizzi

**Affiliations:** ^1^ Infectious Diseases Unit, ASST Papa Giovanni XXIII, Bergamo, Italy; ^2^ Scuola di Medicina, Università degli Studi di Milano-Bicocca, Milano, Italy; ^3^ ATS Bergamo, Ufficio Epidemiologico, Bergamo, Italy; ^4^ Fondazione per la Ricerca Ospedale di Bergamo (FROM)—ETS, Bergamo, Italy; ^5^ Dipartimento di Specialità Medico Chirurgiche, Scienze Radiologiche e Sanità Pubblica, Università Degli Studi di Brescia, Brescia, Italy; ^6^ Central Laboratory, ASST Papa Giovanni XXIII, Bergamo, Italy; ^7^ Scuola di Specializzazione in Radiologia, Università Degli Studi di Milano-Bicocca, Milano, Italy; ^8^ Radiology Unit, ASST Papa Giovanni XXIII, Bergamo, Italy; ^9^ Cardiology Unit, ASST Papa Giovanni XXIII, Bergamo, Italy; ^10^ Immunohematology and Transfusion Medicine, ASST Papa Giovanni XXIII, Bergamo, Italy

**Keywords:** COVID-19, SARS-CoV-2, thrombosis, cardiovascular, vaccination, neutrophils-to-lymphocytes ratio, BRIXIA score

## Abstract

**Importance:**

Population studies have recorded an increased, unexplained risk of post-acute cardiovascular and thrombotic events, up to 1 year after acute severe acute respiratory syndrome coronavirus 2 (SARS-CoV-2) infection.

**Objectives:**

To search for clinical variables and biomarkers associated with late post-acute thrombotic and cardiovascular events after SARS-CoV-2 infection.

**Design:**

Retrospective cohort study.

**Setting:**

Third-level referral hospital in Bergamo (Italy).

**Participants:**

Analysis of an existing database of adult patients, who received care for SARS-CoV-2 infection at our institution between 20 February and 30 September 2020, followed up on a single date (“entry date”) at 3–6 months.

**Exposure:**

Initial infection by SARS-CoV-2.

**Main outcomes and measures:**

Primary outcome: occurrence, in the 18 months after entry date, of a composite endpoint, defined by the International Classification of Diseases—9th edition (ICD-9) codes for at least one of: cerebral/cardiac ischemia, venous/arterial thrombosis (any site), pulmonary embolism, cardiac arrhythmia, heart failure. Measures (as recorded on entry date): history of initial infection, symptoms, current medications, pulmonary function test, blood tests results, and semi-quantitative radiographic lung damage (BRIXIA score). Individual clinical data were matched to hospitalizations, voluntary vaccination against SARS-CoV-2 (according to regulations and product availability), and documented reinfections in the following 18 months, as recorded in the provincial Health Authority database. A multivariable Cox proportional hazard model (including vaccine doses as a time-dependent variable) was fitted, adjusting for potential confounders. We report associations as hazard ratios (HR) and 95% confidence intervals (CI).

**Results:**

Among 1,515 patients (948 men, 62.6%, median age 59; interquartile range: 50–69), we identified 84 endpoint events, occurring to 75 patients (5%): 30 arterial thromboses, 11 venous thromboses, 28 arrhythmic and 24 heart failure events. From a multivariable Cox model, we found the following significant associations with the outcome: previous occurrence of any outcome event, in the 18 months before infection (HR: 2.38; 95% CI: 1.23–4.62); BRIXIA score ≥ 3 (HR: 2.43; 95% CI: 1.30–4.55); neutrophils-to-lymphocytes ratio ≥ 3.3 (HR: 2.60; 95% CI: 1.43–4.72), and estimated glomerular filtration rate < 45 ml/min/1.73 m^2^ (HR: 3.84; 95% CI: 1.49–9.91).

**Conclusions and relevance:**

We identified four clinical variables, associated with the occurrence of post-acute thrombotic and cardiovascular events, after SARS-CoV-2 infection. Further research is needed, to confirm these results.

## Introduction

The risk of thrombosis and acute cardiovascular events (such as heart failure and arrhythmia) is significantly increased in the months after clinical recovery from initial severe acute respiratory syndrome coronavirus 2 (SARS-CoV-2) infection (1–4). This finding comes from large population datasets, mainly based on disease codes or treatment prescriptions, and a more precise clinical characterization is yet to come.

In the early aftermath of the first epidemic wave of SARS-CoV-2 (May–September 2020), we underwent a multidisciplinary follow-up assessment of a large cohort of patients, seen at 3–6 months after acute infection. All of them were directly examined and interviewed in our center and underwent blood tests, chest x-ray (CXR), and pulmonary function tests (PFT).

For each patient, we retrospectively cross-matched the clinical information collected at follow-up, to the occurrence—in the following 18 months—of a composite of prespecified clinical conditions, as recorded in the provincial claims database [this database stores the information about any hospitalization/discharge or emergency room assessment, occurring in private or public hospitals of the Bergamo province, with dates of admission/discharge, and the associated diagnosis, in terms of International Classification of Diseases—9th edition (ICD-9) code (5)]. Our aim was to search for clinical profiles associated with an increased risk of late-onset thrombosis, arrhythmias, and acute cardiovascular events. Reinfections and vaccinations were also considered, as well as the potential occurrence of the same set of clinical conditions in the 18 months prior to COVID-19 onset.

## Methods

### Setting

A third-level referral, public hospital, located in Bergamo (Italy). During the first COVID-19 wave, from 20 February 2020 thereon, our organization was confronted with an unprecedented surge of emergency cases and admissions (6), our region being the first outside of China to be involved in the epidemic.

### Participants

“Surviving COVID” was a public-funded intervention of single time-point follow-up (held from 5 May 2020 to the end of November 2020), offered to all consecutive adult patients, having consulted our Emergency Department (ED) and/or having been admitted to the hospital's wards, with a SARS-CoV-2 infection (confirmed by a molecular test), between 20 February and 30 September 2020. We excluded asymptomatic pregnant women.

The follow-up comprised a general examination and psychological interview, blood tests, CXR, electrocardiogram (ECG), PFT with diffusion, and assessment of rehabilitation needs.

Results of the intervention have been already reported elsewhere (7) (see also Supplementary Material for a detailed description of the enrollment process and the procedures).

The date of the follow-up visit (from 3 to 6 months after the infection) was defined as the *entry date* in this study.

### Data sources

The “Surviving COVID” dataset (containing all clinical variables collected on entry-date) was linked to the existing provincial Health Authority (ATS-BG: Agenzia per la Tutela della Salute, Bergamo) claims database, to retrieve information concerning any hospitalization and ED consultation having occurred in the 18 months after entry date; the available data were: diagnosis—in terms of ICD-9 codes—date of admission/consultation, and date of discharge (if admitted). For comparison, we collected the same information for the 18 months prior to COVID-19 onset. From the same database, we also retrieved all COVID-19 test results (with associated date), and all vaccination doses received (with the product type and associated date), for each patient in the same period.

### Exposures and outcomes

The common exposure was the initial SARS-CoV-2 infection.

### Outcome

Through ICD-9 coding, we established a composite outcome, defined by the occurrence of at least one of the following diagnoses: cerebral/cardiac ischemia, venous/arterial thrombosis (any site), pulmonary embolism, cardiac arrhythmia, heart failure (HF) (see Supplementary Table S1).

Participants were followed from entry-date, until composite outcome occurrence (see below), or removed at death, loss to follow-up, or end of study (fixed at 18 months after entry-date), whichever came first.

### Covariates

Among the clinical information, available from the “Surviving COVID” dataset, we identified all variables possibly and reasonably relevant for the outcome. In particular, we considered:

• the history of the initial infection: age at onset, sex, body mass index (BMI) ≥30 kg/m^2^, income (high vs. intermediate vs. low), hospital admission vs. outpatient treatment, intensive care need, maximal oxygen (O_2_) need attained;
• number and type of preexisting comorbidities (notably: diabetes, cardiovascular or cerebrovascular disease, chronic renal failure, chronic obstructive pulmonary disease, immunosuppression, cirrhosis, neoplasia);
• home therapies at infection onset (notably: anticoagulant, anti-platelet, anti-hypertensive drugs);
• symptoms at follow-up (dyspnea, confusion, asthenia, anosmia, cough);
• PFT at follow-up (single items categorized as pathologic when ≤80% of predicted value);
• blood tests results at follow-up: complete blood count, liver function tests, creatinine and estimated glomerular filtration rate (EGFR) by CKD-EPI algorithm (8), D-dimer, C-reactive protein (CRP), brain natriuretic peptide (BNP); the cutoff for BNP was set at 35 ng/L, as recommended for HF diagnosis, in the 2021 ESC Guidelines (9);
• semi-quantitative radiographic lung damage on follow-up CXR, scored according to BRIXIA score (10, 11). The BRIXIA score of each follow-up CXR was derived through an artificial intelligence algorithm (12), already successfully validated on different datasets (13). Prior validation of the artificial intelligence algorithm on the “Surviving COVID” dataset was obtained, by a double reading of a semi-random choice of 72 CXR (balanced in terms of age ≥ or < 60 years, severity of O_2_ need in acute, and sex), by a couple of radiologists: a senior with more than 10 years of experience, and a resident. The concordance results were satisfying (Supplementary Figures S1, S2);
• eventual SARS-CoV-2 reinfections (defined as a newly positive molecular test, after at least 90 days since the previous positive one);
• the receipt of any dose of vaccine (day of vaccination and type of vaccine);
• occurrence (and date) of any thrombosis or HF or cardiac arrhythmia events (as identified by the same ICD-9 codes as for the composite outcome), in a period of 18 months before initial SARS-CoV-2 infection.

Among the above variables, a list of covariates, in decreasing rank of relevance, was chosen for the multivariable analysis: they were ranked considering at once statistical significance shown in univariate analysis, biological plausibility, and ease of reproducibility in clinical practice (i.e., we favored blood tests to pulmonary functional tests).

### Statistical analysis

Descriptive statistics were used to summarize the characteristics of patients at entry date: number and percentage were reported for categorical variables and mean and standard deviation (SD), or median and interquartile range (IQR), for continuous variables. The chi-square test (or Fisher's exact test when appropriate) was used to test between-group differences for the categorical variables, whereas the *t*-test or the Wilcoxon–Mann–Whitney test (for normally and not normally distributed variables, respectively) was used to compare continuous variables.

Continuous variables were dichotomized, when appropriated, by using the best cutoff identified by the corresponding receiver operating characteristic (ROC) curve.

The Kaplan–Meier estimator was used to estimate the cumulative incidence of the composite outcome.

Univariate and multivariable Cox regression models were fitted to estimate the effect of patients’ characteristics on the risk of composite outcome. The hazard ratios (HRs) and the corresponding 95% confidence intervals (CIs) were reported. The estimates were adjusted for the effect of potential confounders and for vaccine exposure, included as a time-dependent variable. Moreover, a multivariable Fine and Gary competing risk regression model was performed considering death as a competing event.

For all tested hypotheses, two-tailed *p*-values <0.05 were considered significant. Analyses were performed using STATA software, release 16.1 (StataCorp LP, College Station, TX, USA).

### Ethics

Ethics approval was granted from the ASST “Papa Giovanni XXIII” ethical committee (n. 173/21, and amendment n. 61/2022).

A written consent was obtained from all participants at enrollment into the “Surviving COVID” database. All patients had access to the follow-up program regardless of their decision to participate in the study.

## Results

The “Surviving COVID” database counts 1,536 patients, but for 21 of them no linkage was found to the ATS-Bergamo claims database, and they were excluded from the analysis.

The demographic and clinical characteristics of the remaining 1,515 patients (948 men, 62.6%, median age 59; IQR: 50–69) are summarized in the Supplementary Table S2.

During the 18 months of the study, 40 individuals died (2.6%), corresponding to a mortality rate of 0.14% per patient-month (95% CI: 0.10%–0.20%).

In the same period, we identified 84 endpoint events, occurring to 75 patients (5%): 30 arterial thromboses (13 cerebral and 17 coronary), 11 venous thromboses (of which six pulmonary embolism, three phlebitis/superficial thromboses and three deep venous thromboses), 28 arrhythmias and 24 heart failure events (Table 1), corresponding to an incidence rate for the composite outcome of 0.76% per patient-month (95% CI: 0.69–0.83).

**Table 1:** Composite outcome events recorded in the 18 months after entry date (patients could experience more than one event; the number of patients with endpoint occurrence is 75).

	Endpoint events after entry date
	*N* = 84
Thrombotic events	40
Arterial thrombosis (*n* = 30)	
Stroke	11 (13.8)
Transitory ischemic attack	2 (2.5)
Ischemic heart disease	17 (21.3)
Venous thrombosis (*n* = 11)	
Pulmonary embolism	6 (7.5)
Phlebitis and superficial thrombosis	3 (3.8)
Deep venous thrombosis	3 (3.8)
Other events	44
Cardiac arrhythmia	28 (35.0)
Heart failure	24 (30.0)

**Figure 1:**
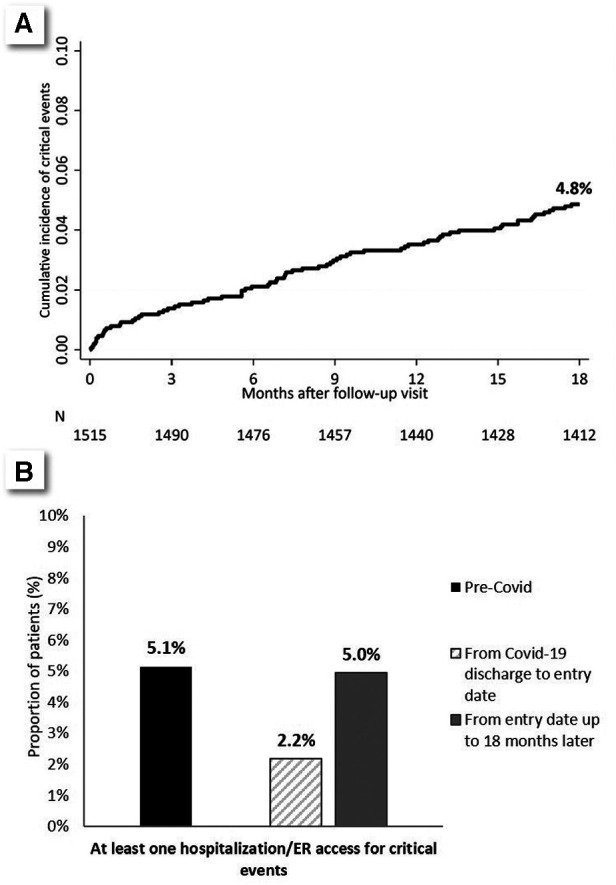
(**A**) Cumulative incidence of composite outcome in the 18 months after entry date. (**B**) Comparison between the prevalence of the composite outcome in the 18 months before infection, in the period from infection to entry date (3–6 months), and in the 18 months after entry date.

The rate did not vary across the 18 months from entry-date (Figure 1A). The frequency of thrombosis/HF/arrhythmic events in the 18 months before COVID-19 was similar to that observed in the 18 months after the entry-date (5.0% vs. 5.1%) (Figure 1B). In particular, the occurrence of thrombosis/HF/cardiac arrhythmia events in the 18 months before SARS-CoV-2 infection was strongly associated with outcome events after entry date: 21.3% vs. 4.3% (*p* < 0.001), with clear segregation between thrombotic events on one side and HF/arrhythmic events on the other (thrombotic events tended to recur in certain patients, and HF/arrhythmic events in others: please refer to Supplementary Table S3, for further details).

Figure 1B also shows the frequency of the events of interest (thrombosis/HF/arrhythmic events) in the immediate post-COVID period (i.e., from hospital discharge to entry date).

Individual characteristics, at COVID-19 onset, significantly associated with the composite outcome were: older age (median age in patients with outcome: 69.0 vs. 59.0, *p* < 0.001), acute-phase hospitalization (81.3% in patients with outcome vs. 68.5%, *p* = 0.019), and preexisting comorbidities (21.3% of patients with outcome had more than two comorbidities vs. 12.1%, *p* < 0.001—see also Supplementary Table S2).

Interestingly, being on treatment with anticoagulant or anti-platelet agents before infection was associated with an increased risk, rather than the opposite. Ischemic or arrhythmic cardiac events during the acute phase, but not thrombotic ones, were predictive of post-follow-up outcome events.

Among variables measured at entry date (follow-up visit) (see Supplementary Tables S4, S5), the ones associated with the outcome were: diffusing capacity of lung for carbon monoxide (DLCO) below 80% of expected (47.5% in patients with outcome vs. 25.6%, *p* < 0.001), BRIXIA score ≥3 (72.9% in patients with outcome vs. 41.4%, *p* < 0.001) and specific blood tests results, notably: D-dimer (median value in patients with the outcome: 466.5 ng/ml vs. 378.0, *p* = 0.003), CRP ≥ 0.5 mg/dl (39.4% in patients with outcome vs. 19.9%, *p* < 0.001), BNP ≥ 35 ng/L (69.6% in patients with outcome vs. 40.7%, *p* < 0.001), and EGFR < 45 ml/min/1.73 m^2^ (17.6% in patients with outcome vs. 3.0%, *p* < 0.001) (see Supplementary Tables S4, S5).

**Figure 2:**
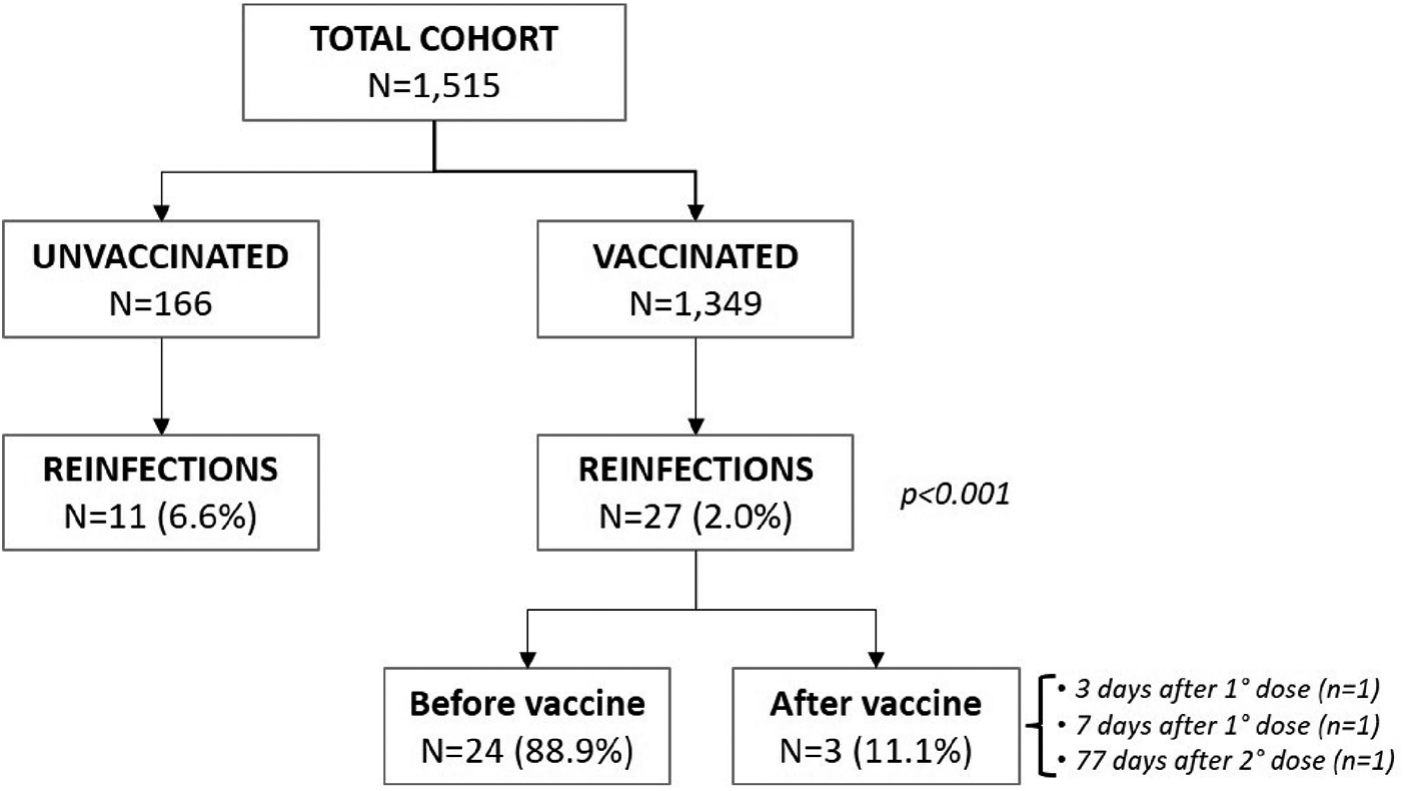
Frequency of reinfections according to vaccine receipt.

Only 38 reinfections during the 18-month follow-up were documented, almost all occurring to non-vaccinated individuals (see Figure 2). From univariate analysis, receipt of any vaccine was associated with a protective effect toward outcome events (among patients incurring in outcome, only 36% were vaccinated vs. 91.8% among those not incurring) not justified by a higher reinfection incidence among unvaccinated (*p* = 0.12) patients. The magnitude of such protective effect was inversely correlated to the number of doses received (*p* < 0.001) (Table 2).

**Table 2:** Comparison of the vaccination and reinfection history among individuals incurring or not in composite outcome events.

	Non-missing (*N*)	Total	Endpoint events after entry date		*p*
		*N* = 1,515	No (*N* = 1,440)	Yes (*N* = 75)	
Vaccine, *n* (%)	1,515	1,349 (89.0)	1,322 (91.8)	27 (36.0)	<0.001
Time in months from COVID-19 to vaccine, median (IQR)			—	9.6 (8.3–10.8)	
Type*, n* (%)	1,349				
Astra Zeneca		233 (17.3)	227 (17.2)	6 (22.2)	0.89
Johnson & Johnson		54 (4.0)	53 (4.0)	1 (3.7)	
Moderna		194 (14.4)	191 (14.4)	3 (11.1)	
Pfizer		868 (64.3)	851 (64.4)	17 (63.0)	
Doses*, n* (%)	1,515				
0		166 (11.0)	118 (8.2)	48 (64.0)	<0.001
1		64 (4.2)	60 (4.2)	4 (5.3)	
2		880 (58.1)	858 (59.6)	22 (29.3)	
3		405 (26.7)	404 (28.1)	1 (1.3)	
Reinfections	1,513	38 (2.5)	34 (2.4)	4 (5.3)	0.12
Months from reinfections to critical events, median (IQR)	4	—	—	3.7 (0.0–7.9)	

“Non-missing (*N*)” indicates the number of observations available.

Results of the Fine and Gray multivariable competing risk model are reported in Table 3. The following variables were significantly associated with the composite outcome:

- previous occurrence of any outcome event in the 18 months before infection [subdistribution HR (sHR): 2.52; 95% CI: 1.27–5.00];
- BRIXIA score ≥3 (sHR: 2.22; 95% CI: 1.22–4.05);
- Neutrophils-to-lymphocytes ratio (NLR) ≥3.3 (sHR: 2.42; 95% CI: 1.32–4.44);
- EGFR <45 ml/min/1.73 m^2^ (sHR: 3.80; 95% CI; 1.42–10.13).

**Table 3:** Multivariable model.

	sHR (95% CI)	*p*
Vaccine	1.33 (0.51–3.45)	0.558
Thrombosis/CV events before COVID-19	2.52 (1.27–5.00)	0.008
Thrombosis during COVID-19 or immediately after (but before entry date)	1.97 (0.95–4.08)	0.069
Male gender	0.78 (0.45–1.35)	0.382
Age at COVID-19 diagnosis	1.00 (0.97–1.02)	0.931
BIXIA at entry date ≥3	2.22 (1.22–4.05)	0.009
BNP at entry date ≥35	2.03 (0.99–4.18)	0.054
NLR at entry date ≥3.3^a^	2.42 (1.32–4.44)	0.004
EGFR at follow-up (ml/min/1.73 m^2^)		
Normal (≥90)	1.00 (reference)	
Mild/moderate (<90 and ≥45)	1.79 (0.85–3.74)	0.123
Severe (<45)	3.80 (1.42–10.13)	0.008

CV, cardiovascular.

^a^ Cutoff identified by the corresponding ROC curve.

To avoid collinearity, we did not include in the multivariable model both CRP and NLR as inflammatory markers, since they were moderately correlated (Pearson's correlation coefficient: 0.40, *p* < 0.05): we chose NLR given its better performance in detecting the outcome of interest (AUC-NLR: 0.66 vs. AUC-PCR: 0.62).

Of note, in the model, both the occurrence of early events (during COVID hospitalization/immediately after discharge but before the entry date) and serum BNP showed a trend toward an increased risk of outcome events (see Table 3).

## Discussion

In a large cohort of individuals, recovering from acute SARS-CoV-2 infection, during the first epidemic wave, we identified four clinical variables independently associated with an increased risk of thrombotic or cardiovascular complications, in the following 18 months.

This was achieved through an integration of structured longitudinal observational data, from our provincial healthcare organization, and direct clinical observations by ourselves (transversally obtained, on a single visit, at 3–6 months from acute SARS-CoV-2 infection). The four clinical variables are (1) a previous history of cardiovascular or thrombotic events, (2) residual radiographic lung involvement (indirectly assessed by a semi-quantitative score, BRIXIA, easily calculated on a posterior-anterior CXR slide), and (3) two laboratory test results, commonly available in clinical routine practice: NLR and EGFR.

We chose the covariates for the multivariable model, according to their recognized association to the outcomes under study, and for their ease of collection, across the large majority of healthcare systems. In fact, our aim was to bring “to the bedside” the epidemiologic observations, coming from the analysis of very big datasets, and characterize the individuals at highest risk, from a “follow-up standpoint.”

The explanation for our findings is not entirely obvious: on one hand, the previous occurrence of a thrombotic and/or cardiac event is a recognized risk for recurrence. Similarly EGFR <45 ml/min/1.73 m^2^ could be regarded as an independent marker of multi-morbidity and/or frailty (14) [that, in their turn, increases the risk of a vast array of cardiovascular events (15)].

On the other side, our finding that an elevated BRIXIA score at follow-up is associated with late-onset thrombotic or cardiovascular events is entirely new. BRIXIA, in fact, has been employed retrospectively to predict mortality from acute COVID-19, displaying a good prognostic value (10, 11, 16). No data exist so far on the dynamics of resolution of CXR abnormalities, following COVID-19, but, for analogy to severe community-acquired pneumonia (17), we suppose that many weeks are necessary. For this reason, an elevated BRIXIA at follow-up could be a proxy for a severe course of the acute infection (we purposely did not include BRIXIA score at the presentation, to be consistent with the “follow-up standpoint” adopted). Seen in this way, our data are in line with others (1–4) who linked the severity of the acute infection to the risk of late-onset thrombotic or cardiovascular outcomes.

The association of late post-COVID events to an increased NLR after recovery is also original and deserves further investigation. NLR is an emerging and promising marker of inflammation (18), associated with adverse outcomes in various conditions, from sepsis (19), to cardiovascular diseases (20–22), and thrombosis in particular (23, 24). It is also a strong predictor of acute COVID-19 severity and outcome (25, 26), that in their turn are associated with SARS-CoV-2-induced activation of platelets, neutrophils, and endothelium (27).

In fact, thrombo-inflammation is a key mechanism in SARS-CoV-2-induced pathology (28), and clinical thrombosis not only complicates acute infection (29) but displays an increased incidence up to 12 months after recovery (longer follow-ups have not been published, so far). Similarly, other inflammatory conditions, such as sepsis (30) or influenza (31), are associated with an increase in cardiovascular complications in the months after resolution, although to a lesser extent: all these conditions could have some common underlying thrombo-inflammatory derangement, yet to be explained. In our study, also CRP showed a significant association with the outcome, in univariate analysis, which reinforces the thrombo-inflammatory hypothesis.

Of note, by no means could any presumed residual inflammation in the post-COVID period be due to viral persistence in the respiratory system, considering that the enrollment in “Surviving COVID” follow-up intervention strictly required confirmed negativity for SARS-CoV-2-RNA on a rhino-pharyngeal swab sample.

Unlike others (1–4), we did not observe an increased incidence of thrombosis, heart failure, or arrhythmia, between pre-COVID and post-follow-up periods (despite a marked increase, during the acute infection phase). Certainly, the sample size considered is too limited, to reveal or exclude an increase of risk of such a limited magnitude.

A strong limitation of our approach is the reliance on ICD-9 codes on the claims database: they were assigned by the various treating physicians without any *a posteriori* revision by us. Given the clinical importance of the outcome events in the study, though, it seems unlikely that any overestimation occurred, while the opposite is still possible.

To overcome the rarity of single-type clinical events, we were forced to adopt a composite outcome approach by grouping together together different cardiovascular conditions, possibly due to independent mechanisms. However, not only are these conditions often grouped similarly in the existing literature (1, 3, 4) on post-COVID conditions, but also, they all likely share a similar thrombo-inflammatory mechanism.

We may have underestimated reinfections because in the period under study, the availability of SARS-CoV-2 tests was extremely limited, and only from health centers: the inverse association between vaccination and outcome events, seen in bivariate but not confirmed in multivariate analysis, could depend on this.

Another strong limitation is that no laboratory or instrumental results, performed on the participants in the pre-COVID period, were available: this prevented a useful comparison to the follow-up results. In fact, since the clinical variables identified are not specific to SARS-CoV-2 infection, it is possible that we simply identified a group of patients at increased risk of thrombosis/cardiovascular events, independently of the post-COVID condition. If this should be the case, though, our observations appear even more valuable, because they suggest once more a role for NLR in risk-stratification for thrombotic/cardiovascular late events after an acute inflammatory insult. In fact, the specific setting of our study (the recent and first-ever exposition to an entirely new and highly virulent pathogen, on a relatively circumscribed population, and over a short time period) appears poorly transferrable to the current SARS-CoV-2 clinical burden (with newer variants of reduced pathogenicity). Nonetheless, for the same reasons, the cohort that we studied (large, treated in a single center, re-evaluated in-person, and in a standardized intervention) provides data from a unique “experiment of nature,” about the long-term effects on cardiovascular health, entailed by acute infection.

## Conclusions

The aim of this study was to search for relevant clinical variables associated with late-onset post-COVID thrombosis and acute cardiovascular events, as a step toward a better understanding of the post-COVID recovery process. We identified four variables, very simple to obtain from patients, on a follow-up visit, even though their role as prognostic tools deserves validation on larger datasets. Thanks to their non-specificity, our results (in particular the finding of an increased NLR as associated with cardiovascular outcomes) appear as potentially transferrable (after proper testing and validation) to the follow-up of patients recovering from non-COVID pneumonia, bacterial sepsis or other severe infections.

## Data Availability

The raw data supporting the conclusions of this article will be made available by the authors, without undue reservation.
