# Supplementary material for: Clinical variables associated with late-onset thrombotic and cardiovascular events, after SARS-CoV-2 infection, in a cohort of patients from the first epidemic wave: an 18-month analysis on the “Surviving-COVID” cohort from Bergamo, Italy
Source: Front Cardiovasc Med. 2023 Nov 30;10:1280584. doi: 10.3389/fcvm.2023.1280584 (PMC10720075; doi:10.3389/fcvm.2023.1280584)
Supplement: Supplementary file 1 [file Datasheet1.docx]

Supplementary Material

**SUPPLEMENTARY MATERIAL**

**Index of contents:**

1. “Surviving COVID” intervention: enrolment and procedures
2. Composite Outcome definition: ICD-9 codes considered (Table-S1)
3. Acute infection details (Table-S2) and univariate associations with the outcome
4. Composite Outcome events recurrence comparing pre-COVID and post-entry-date periods (Table-S3)
5. Follow-up findings (eTable-4) and univariate associations with the outcome
6. Blood tests results at follow-up and univariate associations with the outcome (eTable-5)
7. BRIXIA score description
8. Concordance of BRIXIA score readings, among human eye and Artificial Intelligence on a validation sample from “Surviving COVID” dataset (eFigure-1 and -2)

**1 - “Surviving COVID” intervention: enrolment and procedures**

Patients still in-hospital were put on a ‘waiting list’, and recalled once discharged. Enrolment was on a voluntary basis only after a double-negative nasopharyngeal swab for SARS-CoV-2 RNA, as required at that time by the Italian Health Authority. For cognitively impaired subjects, a caregiver helped in providing information about the medical history and in recalling the pre-acute episode health status.

The intervention was two-step:

• Step 1: nurse-led evaluation with vital signs assessment, height and weight measurement, comprehensive blood tests, chest-X-ray (CXR), electrocardiogram (ECG), full pulmonary function testing with diffusion, psychological evaluation, assessment of rehabilitation needs.

PFT were performed according to current standards [1] by professionally trained respiratory technicians using Medical Graphics Elite Pro body box equipped with rapid gas analysers (MGC Diagnostics Corporation, USA) and interpreted by two experienced pulmonologists following current recommendations [2]. On account of COVID-19 restrictions, PFT were limited to spirometry and diffusing capacity for carbon monoxide (DLCO) [3]. Spirometric parameters comprised alveolar volume (VA), carbon monoxide transfer coefficient (KCO), forced vital capacity (FVC), forced expiratory volume in the first second (FEV1), and FEV1/FVC ratio. PFT parameters were expressed as a percent of the predicted value (%) and considered impaired if below the lower limit of normal according to the Global Lung Function Initiative 2012 reference equations for spirometry [4] and the Global Lung Function Initiative 2017 reference equations for DLCO [5].

• Step 2 (three days later): infectious diseases consultation and, if appropriate, subsequent referral to primary care or to other specialists.

Several assessment scales were adopted at Step 1, and their results were discussed with the patients at the end of the clinical psychological interview. In particular:

- For assessment of rehabilitation needs we adopted the Barthel Index, as a measure of the individual disability [12], and the Brief Fatigue Inventory (BFI) scale, as a measure of the level of fatigue [13]. Conditions pre-existing to the acute COVID-19 episode were also scored using these two scales, by asking patients to recall their symptoms
- For psychological evaluation, self-report questionnaires were administered: in particular, to evaluate post-traumatic stress disorder (PTSD), we employed Impact of Events Scale - Revised (IES-R) [14].
- SF-36 was employed to measure the health-related quality of life (HR-QoL) [15]

In addition, on Step 1 each patient received a paper form, to be filled at home and returned: this was the so-called “Socio-economical Questionnaire” (SQ), enquiring about the socio-economical characteristics of the patient (mainly: level of study, working condition, number of minors in the household). Country of birth was taken as a proxy for foreign origin. SQ collected also information about “how many people in the patient’s household had been admitted for COVID”, and “how many in the 1st degree familiars had died because of COVID”.

On step 2 a complete review of the clinical history of the acute COVID-19 episode was undertaken, and presence of the following comorbidities recorded: diabetes, hypertension, atrial fibrillation, previous myocardial infarction or revascularization, previous stroke, previous cardiac decompensation, Chronic Obstructive Pulmonary Disease (COPD), active malignancy (hematologic or solid), autoimmune disease.

Admission to hospital wards or intensive care units (ICUs) was tracked, as well as maximal O2 support attained during the admission (as a proxy for the clinical severity).

We also recorded cigarette smoking (present or past), as declared by patients, date of onset of symptoms (as remembered on the day of the follow-up visit), first date of hospital consultation with a confirmed SARS-CoV-2 infection, and date of hospital admission, if applicable.

REFERENCES

1. Graham B.L., Steenbruggen I., Miller M.R., Barjaktarevic I.Z., Cooper B.G., Hall G.L., Hallstrand T.S., Kaminsky D.A., McCarthy K., McCormack M.C., Oropez C.E., Rosenfeld M., Stanojevic S., Swanney M.P., Thompson B.R.: Standardization of spirometry 2019 update. An Official American Thoracic Society and European Respiratory Society technical statement. Am. J. Respir. Crit. Care Med. 2019; 200: pp. e70-e88.

2. Pellegrino R., Viegi G., Brusasco V., Crapo R.O., Burgos F., Casaburi R., Coates A., van der Grinten C.P., Gustafsson P., Hankinson J., Jensen R., Johnson D.C., MacIntyre N., McKay R., Miller M.R., Navajas D., Pedersen O.F., Wanger J.: Interpretative strategies for lung function tests. Eur. Respir. J. 2005; 26: pp. 948-968.

3. McGowan A., Sylvester K., Burgos F., Boros P., de Jongh F., Kendrick A., Lloyd Cooper J., Kirkby J., Makonga-Braaskma J., Steenbruggen I., den Berg J.: ERS 9.1 Statement on lung function during COVID-19 Final with Contributors. Ers 2020; pp. 1-5. Accessed August 2, 2020 https://ers.app.box.com/s/zs1uu88wy51monr0ewd990itoz4tsn2h

4. Quanjer P.H., Stanojevic S., Cole T.J., Baur X., Hall G.L., Culver B.H., Enright P.L., Hankinson J.L., Ip M.S., Zheng J., Stocks J.: ERS Global Lung Function Initiative. Multi-ethnic reference values for spirometry for the 3-95-yr age range: the global lung function 2012 equations. Eur. Respir. J. 2012; 40: pp. 1324-1343.

5. Stanojevic S., Graham B.L., Cooper B.G., Thompson B.R., Carter K.W., Francis R.W., Hall G.L.: Global Lung Function Initiative TLCO working group; Global Lung Function Initiative (GLI) TLCO. Official ERS technical standards: Global Lung Function Initiative reference values for the carbon monoxide transfer factor for Caucasians. Eur. Respir. J. 2017; 50:

**2 - Table-S1: Composite Outcome definition: ICD-9 codes considered**

| **Diagnosis** | **ICD-9 codes** |
| --- | --- |
| ischemic stroke | 433-434 |
| transitory ischemic attack | 435 |
| ischemic heart disease | 410-414 |
| venous thrombosis (other) | 325, 336.1, 453, 671.2-5, 999.2 |
| pulmonary embolism | 415 |
| arterial thrombosis and embolism | 440.3, 444-445 |
| retinal thrombosis | 362.3 |
| mesenteric thrombosis | 557 |
| portal thrombosis | 452 |
| phlebitis and superficial thrombosis | 451 |
| renal artery thrombosis | 593.81 |
| penis thrombosis | 607.82 |
| cardiac arrhythmia | 427 |
| heart failure | 428 |

**3 - Table-S2: Acute infection details and univariate associations with the outcome**

|  | **N non missing** | **Total** | **CRITICAL EVENTS POST-FUP** | | ***p*** |
| --- | --- | --- | --- | --- | --- |
|  |  | **N=1,515** | **NO (N=1,440)** | **YES (N=75)** |  |
| **Age at Covid-19 diagnosis**, median (IQR) | 1,513 | 59.0 (50.0-69.0) | 59.0 (50.0-68.0) | 68.0 (60.0-75.0) | <0.001 |
| **Male gender**, *n (%)* | 1,515 | 948 (62.6) | 902 (62.6) | 46 (61.3) | 0.82 |
| **Critical events (pre-COVID)**, *n (%)* | 1,515 | 78 (5.1) | 62 (4.3) | 16 (21.3) | <0.001 |
| **Wave**, *n (%)* | 1,513 |  |  |  |  |
| 1 (wild-type) |  | 1,492 (98.6) | 1,417 (98.5) | 75 (100.0) | 0.62 |
| 2 (alpha, beta, gamma) |  | 21 (1.4) | 21 (1.5) | 0 (0.0) |  |
| **Income** | 1,228 |  |  |  |  |
| Low |  | 306 (24.9) | 292 (24.9) | 14 (25.0) | 0.33 |
| Median |  | 445 (36.2) | 420 (35.8) | 25 (44.6) |  |
| High |  | 477 (38.8) | 460 (39.2) | 17 (30.4) |  |
| **Hospitalization for Covid-19** | 1,515 | 1,048 (69.2) | 987 (68.5) | 61 (81.3) | 0.019 |
| **BMI ≥ 30** (Kg/m^2^) | 1,178 | 252 (21.4) | 238 (21.4) | 14 (20.6) | 0.87 |
| **Respiratory support**, *n (%)* | 1,513 |  |  |  |  |
| None |  | 582 (38.5) | 563 (39.2) | 19 (25.3) | 0.051 |
| Non-invasive |  | 592 (39.1) | 555 (38.6) | 37 (49.3) |  |
| Invasive |  | 339 (22.4) | 320 (22.3) | 19 (25.3) |  |
| **ICU hospitalization** | 1,515 | 149 (9.8) | 138 (9.6) | 11 (14.7) | 0.15 |
| **Brixia Score on the 1^st^ CXR available** | 1,100 | 5.0 (2.0-8.0) | 5.0 (2.0-8.0) | 6.0 (4.0-9.0) | 0.027 |
| **≥** 8 | 1,100 | 308 (28.0) | 287 (27.6) | 21 (33.9) | 0.29 |
| **N° Comorbidities**, *n (%)* | 1,515 |  |  |  |  |
| 0 |  | 836 (55.2) | 813 (56.5) | 23 (30.7) | <0.001 |
| 1 |  | 489 (32.3) | 453 (31.5) | 36 (48.0) |  |
| 2+ |  | 190 (12.5) | 174 (12.1) | 16 (21.3) |  |
| **Diabetes** | 1,515 | 175 (11.6) | 160 (11.1) | 15 (20.0) | 0.019 |
| **Cardiovascular disease** | 1,515 | 557 (36.8) | 515 (35.8) | 42 (56.0) | <0.001 |
| **Chronic renal failure** | 1,515 | 92 (6.1) | 78 (5.4) | 14 (18.7) | <0.001 |
| **COPD** | 1,515 | 52 (3.4) | 44 (3.1) | 8 (10.7) | <0.001 |
| **Autoimmune disease** | 1,515 | 57 (3.8) | 50 (3.5) | 7 (9.3) | 0.009 |
| **Neoplasms** | 1,515 | 68 (4.5) | 64 (4.4) | 4 (5.3) | 0.72 |
| **Liver cirrhosis** | 1,515 | 8 (0.5) | 8 (0.6) | 0 (0.0) | 0.52 |
| **Immunosuppression** | 1,515 | 20 (1.3) | 19 (1.3) | 1 (1.3) | 0.99 |
| **Cerebrovascular disease** | 1,515 | 44 (2.9) | 37 (2.6) | 7 (9.3) | <0.001 |
| **Home therapies**, *n (%)* |  |  |  |  |  |
| ACEs | 1,515 | 187 (12.3) | 171 (11.9) | 16 (21.3) | 0.015 |
| ARBs | 1,515 | 192 (12.7) | 180 (12.5) | 12 (16.0) | 0.37 |
| Other antihypertensives | 1,515 | 370 (24.4) | 333 (23.1) | 37 (49.3) | <0.001 |
| Steroids | 1,515 | 43 (2.8) | 36 (2.5) | 7 (9.3) | <0.001 |
| Antidiabetics | 1,515 | 125 (8.3) | 116 (8.1) | 9 (12.0) | 0.23 |
| Insulin | 1,515 | 39 (2.6) | 33 (2.3) | 6 (8.0) | 0.002 |
| inhalers | 1,515 | 57 (3.8) | 54 (3.8) | 3 (4.0) | 0.91 |
| Oral anticoagulants | 1,515 | 80 (5.3) | 66 (4.6) | 14 (18.7) | <0.001 |
| Antiplatelet agents | 1,515 | 208 (13.7) | 180 (12.5) | 28 (37.3) | <0.001 |
| Proton pump inhibitors | 1,515 | 253 (16.7) | 223 (15.5) | 30 (40.0) | <0.001 |
| **Complications**, *n (%)* |  |  |  |  |  |
| Pneumological | 1,515 | 124 (8.2) | 114 (7.9) | 10 (13.3) | 0.095 |
| Cardio-arrhythmic | 1,515 | 61 (4.0) | 46 (3.2) | 15 (20.0) | <0.001 |
| Cardio-ischemic | 1,515 | 21 (1.4) | 17 (1.2) | 4 (5.3) | 0.003 |
| Cardio-inflammatory | 1,515 | 24 (1.6) | 21 (1.5) | 3 (4.0) | 0.086 |
| Nephrological | 1,515 | 25 (1.7) | 17 (1.2) | 8 (10.7) | <0.001 |
| Thrombotic | 1,515 | 89 (5.9) | 81 (5.6) | 8 (10.7) | 0.070 |
| haemorrhagic | 1,515 | 21 (1.4) | 17 (1.2) | 4 (5.3) | 0.003 |
| Neuropsychiatric | 1,515 | 40 (2.6) | 36 (2.5) | 4 (5.3) | 0.14 |
| Neurological | 1,515 | 50 (3.3) | 46 (3.2) | 4 (5.3) | 0.31 |
| Neurological (peripheral) | 1,515 | 48 (3.2) | 44 (3.1) | 4 (5.3) | 0.27 |
| Infectious | 1,515 | 61 (4.0) | 53 (3.7) | 8 (10.7) | 0.003 |
| Immuno-rheumatological | 1,515 | 33 (2.2) | 32 (2.2) | 1 (1.3) | 0.61 |

**4 - Table-S3: Composite Outcome events recurrence comparing pre-COVID and post-entry-date periods**

|  | **THROMBOSIS AFTER ENTRY-DATE** | | ***p*** |
| --- | --- | --- | --- |
|  | **NO (N=1,475)** | **YES (N=40)** |  |
| Thrombosis before COVID | 46 (3.1) | 4 (10.0) | 0.016 |

|  | **HF/ARRHYTHMIA AFTER ENTRY-DATE** | | ***p*** |
| --- | --- | --- | --- |
|  | **NO (N=1,471)** | **YES (N=44)** |  |
| HF/ arrhythmia before COVID | 26 (1.8) | 10 (22.7) | <0.001 |

**5 - Table-S4: Follow-up findings and univariate associations with the outcome**

|  | **N non missing** | **Total** | **CRITICAL EVENTS POST-FUP** | | ***p*** |
| --- | --- | --- | --- | --- | --- |
|  |  | **N=1,515** | **NO (N=1,440)** | **YES (N=75)** |  |
| **Age at follow-up visit** | 1,515 | 60.0 (51.0-69.0) | 59.0 (50.0-68.0) | 69.0 (60.0-75.0) | <0.001 |
| **Days from Covid-19** (discharge date, if hospitalized, or symptoms onset, if home-treated), median (IQR) | 1,513 | 106.0 (70.0-141.0) | 107.0 (71.0-141.0) | 84.0 (65.0-123.0) | 0.008 |
| **Smoke**, *n (%)* |  |  |  |  |  |
| *Former smoker* | 1,515 | 373 (24.6) | 351 (24.4) | 22 (29.3) | 0.33 |
| *Current smoker* | 1,515 | 82 (5.4) | 80 (5.6) | 2 (2.7) | 0.28 |
| **N° symptoms complained**, *n (%)* | 1,515 | 1.0 (0.0-1.0) | 1.0 (0.0-1.0) | 1.0 (0.0-2.0) | 0.022 |
| *None* | 1,515 | 699 (46.1) | 669 (46.5) | 30 (40.0) | 0.27 |
| *Dyspnoea* | 1,515 | 309 (20.4) | 291 (20.2) | 18 (24.0) | 0.43 |
| *Fever* | 1,515 | 5 (0.3) | 3 (0.2) | 2 (2.7) | 0.022 |
| *Cough* | 1,515 | 47 (3.1) | 41 (2.8) | 6 (8.0) | 0.012 |
| *Confusion* | 1,515 | 34 (2.2) | 29 (2.0) | 5 (6.7) | 0.008 |
| *Asthenia* | 1,515 | 492 (32.5) | 466 (32.4) | 26 (34.7) | 0.68 |
| *myalgia* | 1,515 | 79 (5.2) | 68 (4.7) | 11 (14.7) | <0.001 |
| *Anosmia* | 1,515 | 61 (4.0) | 58 (4.0) | 3 (4.0) | 0.99 |
| *Headache* | 1,515 | 14 (0.9) | 13 (0.9) | 1 (1.3) | 0.51 |
| *Syncope* | 1,515 | 2 (0.1) | 2 (0.1) | 0 (0.0) | 1.00 |
| *Palpitations* | 1,515 | 45 (3.0) | 40 (2.8) | 5 (6.7) | 0.053 |
| *Chest pain* | 1,515 | 56 (3.7) | 55 (3.8) | 1 (1.3) | 0.27 |
| *Gastrointestinal (upper)* | 1,515 | 5 (0.3) | 4 (0.3) | 1 (1.3) | 0.22 |
| *Gastrointestinal (lower)* | 1,515 | 16 (1.1) | 15 (1.0) | 1 (1.3) | 0.56 |
| *Other* | 1,515 | 144 (9.5) | 139 (9.7) | 5 (6.7) | 0.39 |
| **Current therapies,** *n (%)* |  |  |  |  |  |
| Antibiotics | 1,515 | 1 (0.1) | 1 (0.1) | 0 (0.0) | 1.00 |
| Antithrombotic | 1,515 | 22 (1.5) | 21 (1.5) | 1 (1.3) | 0.93 |
| Anticoagulants | 1,515 | 98 (6.5) | 88 (6.1) | 10 (13.3) | 0.013 |
| Steroids | 1,515 | 28 (1.8) | 25 (1.7) | 3 (4.0) | 0.16 |
| O2 (LTOT) | 1,515 | 8 (0.5) | 7 (0.5) | 1 (1.3) | 0.33 |
| Hydroxychloroquine | 1,515 | 2 (0.1) | 2 (0.1) | 0 (0.0) | 1.00 |
| HIV-inhibitors | 1,515 | 1 (0.1) | 1 (0.1) | 0 (0.0) | 1.00 |
| Other | 1,515 | 98 (6.5) | 92 (6.4) | 6 (8.0) | 0.58 |
| **Pulmonary function parameters** |  |  |  |  |  |
| FEV1 (L)*, median (IQR)* | 1,446 | 3.0 (2.5-3.7) | 3.1 (2.5-3.7) | 2.4 (1.9-3.3) | <0.001 |
| FEV1 (%)*, median (IQR)* | 1,445 | 100.1 (88.7-110.4) | 100.6 (89.2-110.4) | 91.2 (75.4-108.3) | 0.001 |
| FVC (L)*, median (IQR)* | 1,446 | 3.8 (3.1-4.6) | 3.8 (3.1-4.6) | 3.1 (2.3-4.0) | <0.001 |
| FVC (%)*, median (IQR)* | 1,447 | 97.8 (86.6-108.0) | 98.0 (86.9-108.2) | 91.0 (72.0-102.2) | 0.001 |
| DLCO (L)*, median (IQR)* | 1,382 | 23.4 (18.2-28.7) | 23.7 (18.5-28.9) | 18.6 (12.6-23.6) | <0.001 |
| DLCO <80%, *n (%)* | 1,376 | 366 (26.6) | 337 (25.6) | 29 (47.5) | <0.001 |
| **Brixia Score***, median (IQR)* | 1,145 | 2.0 (1.0-4.0) | 2.0 (1.0-4.0) | 4.0 (2.0-6.0) | <0.001 |
| **Brixia ≥ 3**, *n (%)* | 1,145 | 493 (43.1) | 450 (41.4) | 43 (72.9) | <0.001 |

**6 - Table-S5: Blood tests results at follow-up and univariate associations with the outcome**

|  | **N non missing** | **Total** | **CRITICAL EVENTS POST-FUP** | | **p** |
| --- | --- | --- | --- | --- | --- |
|  |  | **N=1,515** | **NO (N=1,440)** | **YES (N=75)** |  |
| **HB** (g/L) | 1,511 | 141.0 (130.0-151.0) | 141.0 (131.0-151.0) | 129.0 (117.0-144.0) | <0.001 |
| **HCT** (%) | 1,511 | 41.4 (38.5-44.0) | 41.4 (38.7-44.0) | 38.7 (35.9-42.9) | <0.001 |
| **PLT** (10^9^/L) | 1,508 | 231.0 (196.0-271.0) | 232.0 (197.0-270.0) | 225.0 (182.0-290.0) | 0.89 |
| **WBC** (10^9^/L) | 1,511 | 6.4 (5.4-7.6) | 6.4 (5.4-7.6) | 7.2 (6.0-8.3) | 0.001 |
| **Neutrophils** (10^9^/L) | 1,511 | 3.5 (2.8-4.4) | 3.5 (2.7-4.3) | 4.1 (3.4-5.6) | <0.001 |
| **Lymphocytes** (10^9^/L) | 1,506 | 2.0 (1.6-2.5) | 2.0 (1.6-2.5) | 1.9 (1.3-2.3) | 0.008 |
| **N/L ratio** | 1,506 | 1.7 (1.3-2.3) | 1.7 (1.3-2.3) | 2.0 (1.5-3.8) | <0.001 |
| **N/L ratio ≥ 3.3**, *n (%)* | 1,506 | 151 (10.0) | 128 (8.9) | 23 (31.1) | <0.001 |
| **RBC** (10^12^/L) | 1,511 | 4.7 (4.4-5.1) | 4.7 (4.4-5.1) | 4.4 (4.0-4.9) | <0.001 |
| **RDW** (%) | 1,511 | 13.2 (12.6-14.0) | 13.2 (12.5-13.9) | 14.2 (13.0-15.3) | <0.001 |
| **RDW >14.5%**, *n (%)* | 1,511 | 256 (16.9) | 228 (15.9) | 28 (37.8) | <0.001 |
| **MCV** (fL) | 1,511 | 87.8 (84.9-90.7) | 87.7 (84.8-90.5) | 90.3 (85.9-92.4) | <0.001 |
| **Urea** (mg/dL) | 1,510 | 39.0 (32.0-47.0) | 39.0 (32.0-46.0) | 44.0 (34.0-69.0) | <0.001 |
| **Glucose** (mg/dL) | 1,423 | 86.0 (80.0-95.0) | 86.0 (80.0-95.0) | 88.5 (79.0-99.5) | 0.24 |
| **Total bilirubin** (mg/dL) | 1,495 | 0.7 (0.5-1.0) | 0.7 (0.5-1.0) | 0.7 (0.5-1.0) | 0.38 |
| **Direct bilirubin** (mg/dL) | 316 | 0.4 (0.3-0.5) | 0.4 (0.3-0.5) | 0.5 (0.4-0.6) | 0.072 |
| **Protein** (g/dL) | 1,502 | 6.7 (6.5-7.0) | 6.7 (6.5-7.0) | 6.6 (6.3-7.0) | 0.006 |
| **EGFR** (mL/min 1.73m^2^) | 1,508 | 92.0 (81.0-101.5) | 92.5 (82.0-101.9) | 78.3 (52.4-91.1) | <0.001 |
| Normal/high (≥ 90) |  | 850 (56.4) | 828 (57.7) | 22 (29.7) | <0.001 |
| Mild/ Moder.(< 90 and ≥ 45) |  | 602 (39.9) | 563 (39.3) | 39 (52.7) |  |
| Severe/Kidney failure (< 45) |  | 56 (3.7) | 43 (3.0) | 13 (17.6) |  |
| **AST** (U/L) | 1,502 | 20.0 (16.0-25.0) | 20.0 (16.0-25.0) | 20.0 (17.0-23.0) | 0.99 |
| **ALT** (U/L) | 1,505 | 24.0 (18.0-33.0) | 24.0 (18.0-33.0) | 21.5 (17.0-29.0) | 0.033 |
| **GGT** (U/L) | 1,508 | 24.0 (17.0-35.0) | 23.0 (17.0-35.0) | 28.0 (20.0-48.0) | 0.021 |
| **LDH** (U/L) | 1,505 | 211.0 (189.0-236.0) | 211.0 (188.0-234.0) | 228.5 (203.0-253.0) | <0.001 |
| **Albumin%** | 1,509 | 61.7 (58.9-64.0) | 61.8 (59.1-64.0) | 59.3 (55.5-62.4) | <0.001 |
| **Alpha-1%** | 1,509 | 3.7 (3.3-4.1) | 3.6 (3.3-4.1) | 4.2 (3.7-5.3) | <0.001 |
| **Alpha-2%** | 1,509 | 9.2 (8.1-10.3) | 9.1 (8.1-10.3) | 10.2 (8.8-12.2) | <0.001 |
| **Gamma%** | 1,509 | 14.3 (12.5-16.4) | 14.3 (12.5-16.4) | 14.3 (12.5-16.3) | 0.71 |
| **Beta-1%** | 1,509 | 5.8 (5.5-6.2) | 5.8 (5.5-6.2) | 5.8 (5.4-6.5) | 0.54 |
| **Beta-2%** | 1,509 | 5.0 (4.5-5.7) | 5.0 (4.4-5.6) | 5.3 (4.7-5.8) | 0.080 |
| **Albumin** (g/dL) | 1,502 | 4.1 (3.9-4.3) | 4.1 (4.0-4.3) | 3.9 (3.7-4.1) | <0.001 |
| **Alpha-1** (g/dL) | 1,502 | 0.2 (0.2-0.3) | 0.2 (0.2-0.3) | 0.3 (0.2-0.3) | <0.001 |
| **Alpha-2** (g/dL) | 1,502 | 0.6 (0.6-0.7) | 0.6 (0.5-0.7) | 0.7 (0.6-0.8) | <0.001 |
| **Gamma** (g/dL) | 1,502 | 0.9 (0.8-1.1) | 0.9 (0.8-1.1) | 0.9 (0.8-1.1) | 0.64 |
| **Albumin/Gamma** | 1,509 | 1.6 (1.4-1.8) | 1.6 (1.4-1.8) | 1.5 (1.3-1.7) | <0.001 |
| **Beta-2** (g/dL) | 1,502 | 0.3 (0.3-0.4) | 0.3 (0.3-0.4) | 0.3 (0.3-0.4) | 0.69 |
| **Beta-1** (g/dL) | 1,502 | 0.4 (0.4-0.4) | 0.4 (0.4-0.4) | 0.4 (0.3-0.4) | 0.39 |
| **TSH** (µU/mL) | 1,394 | 1.6 (1.0-2.2) | 1.6 (1.0-2.2) | 1.6 (1.1-2.5) | 0.82 |
| **ATPO** (UI/mL) | 1,273 | 41.0 (33.0-52.0) | 41.0 (33.0-52.0) | 37.0 (30.0-46.0) | 0.025 |
| **ABTG** (UI/mL) | 1,024 | 20.0 (16.0-29.0) | 20.0 (16.0-28.0) | 19.0 (0.0-32.0) | 0.68 |
| **D-Dimer** (ng/mL) | 1,326 | 382.0 (261.0-651.0) | 378.0 (259.0-638.0) | 466.5 (305.5-1061.0) | 0.003 |
| <500 |  | 862 (65.0) | 826 (65.7) | 36 (52.9) | 0.009 |
| 500-1000 |  | 277 (20.9) | 263 (20.9) | 14 (20.6) |  |
| ≥1000 |  | 187 (14.1) | 169 (13.4) | 18 (26.5) |  |
| **INR** | 1,483 | 1.0 (0.9-1.0) | 1.0 (0.9-1.0) | 1.0 (1.0-1.2) | <0.001 |
| **CRP** (mg/dL) | 1,223 | 0.1 (0.1-0.4) | 0.1 (0.1-0.3) | 0.3 (0.1-1.2) | <0.001 |
| ≥0.5 |  | 256 (20.9) | 230 (19.9) | 26 (39.4) | <0.001 |
| **HBA1C** (mmol/mol) | 1,393 | 32.0 (5.6-40.0) | 32.0 (5.6-40.0) | 7.7 (5.6-40.0) | 0.92 |
| **Urine: Albumin/creatinine** | 1,062 | 3.0 (2.0-7.0) | 3.0 (2.0-7.0) | 9.0 (4.0-46.0) | <0.001 |
| <10 |  | 861 (81.1) | 837 (82.5) | 24 (51.1) | <0.001 |
| 10-30 |  | 115 (10.8) | 108 (10.6) | 7 (14.9) |  |
| 30-300 |  | 73 (6.9) | 59 (5.8) | 14 (29.8) |  |
| 300+ |  | 13 (1.2) | 11 (1.1) | 2 (4.3) |  |
| **BNP** (ng/L) | 1,382 | 27.5 (14.0-58.0) | 26.0 (13.0-55.0) | 82.0 (31.0-239.0) | <0.001 |
| **BNP ≥35 ng/L**, *n (%)* | 1,382 | 583 (42.2) | 535 (40.7) | 48 (69.6) | <0.001 |
| **Pre-albumin** (mg/L) | 1,394 | 272.0 (238.0-307.0) | 273.0 (239.0-307.0) | 246.0 (208.0-283.0) | <0.001 |

**7- BRIXIA score description**

Briefly, the anterior chest radiogram is divided into 6 sub-zones and the disease involvement of each is semi-quantitatively scored from 0 to 3, where 0 is a normal radio-transparency. A synthetic global score is evaluated by summing the 6 regional scores with a 0-18 dynamic, and a global score ≥ 8 has appears as the best cut-off for the prediction of in-hospital mortality in acute^[[1]](#footnote-1)^.

**8 - Figure-S1, Figure-S2: Concordance of BRIXIA score readings, among human eye and Artificial Intelligence on a validation sample from “Surviving COVID” dataset**


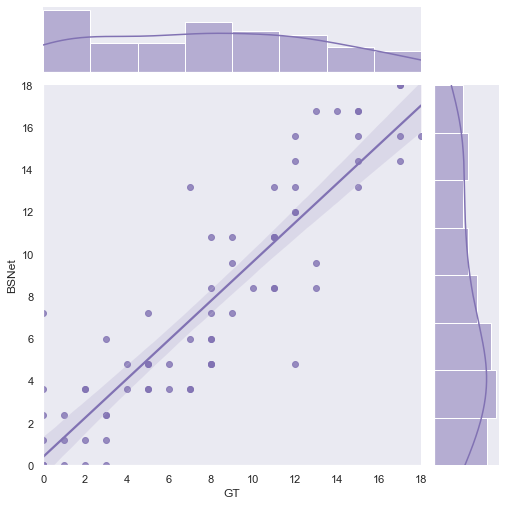


eFigure-1: Validation sample result for the BRIXIA Score Artificial Intelligence algorithm (results from 0 to 18). Seventy-two CXR from onset evaluation were semi-randomly chosen (assuring a balanc in terms of age ≥ or < 60 years, severity of O2 need in acute, and sex), and read in parallel by a couple of radiologists: a senior with more than ten years of experience, and a resident.

(GT: radiologists’ read results - BSNet: AI algorithm read results).

Mean Absolute Error = 1.85 (std 1.55).


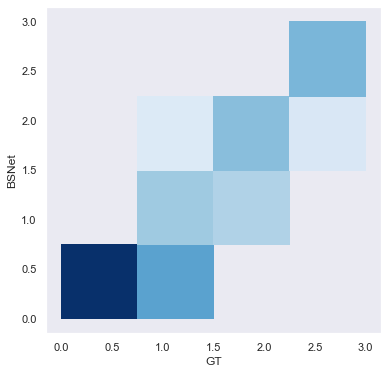


eFigure-2: Here, the graphic shows the concordance among the according to the lung region considered.

(GT: radiologists’ read results - BSNet: AI algorithm read results).

Mean Absolute Error = 0.47 (std 0.61).

1. https://brixia.github.io/ [↑](#footnote-ref-1)
